# Inflammatory Responses to Monomeric and Aggregated α-Synuclein in Peripheral Blood of Parkinson Disease Patients

**DOI:** 10.3389/fnins.2021.639646

**Published:** 2021-03-25

**Authors:** Federica Piancone, Marina Saresella, Francesca La Rosa, Ivana Marventano, Mario Meloni, Jorge Navarro, Mario Clerici

**Affiliations:** ^1^Laboratory of Molecular Medicine and Biotechnology, IRCCS Fondazione don C Gnocchi, Milan, Italy; ^2^Department of Neurology, IRCCS Fondazione don C Gnocchi, Milan, Italy; ^3^Department of Physiopathology and Transplants, University of Milano, Milan, Italy

**Keywords:** Parkinson’s disease, α-synuclein, inflammation, inflammasome, cytokines

## Abstract

To investigate whether different forms of α-synuclein (α-syn) proteins can induce inflammation and activate the NLRP3 inflammasome, we stimulated with monomeric or aggregated α-syn peripheral blood mononuclear cells of Parkinson disease (PD) patients and age- and sex-matched healthy controls (HC). ASC-speck formation, i.e., the intracellular generation of functionally active inflammasome complexes, as well as the production of inflammasome-related [caspase-1, interleukin 1β (IL-18), and IL-1β], and pro–IL-6, or anti–IL-10 inflammatory cytokines were evaluated. Gastrointestinal permeability, suggested to be altered in PD, was also investigated by measuring plasma concentration of lipopolysaccharide (LPS) and I-FABP (fatty acid–binding protein). ASC-speck expression, as well as IL-18 and caspase-1 production and LPS and I-FABP plasma concentration, was comparable in PD and HC, indicating that α-syn does not stimulate the NLRP3 inflammasome and that PD does not associate with alterations of intestinal permeability. Interestingly, though, IL-1β and IL-6 production was increased, whereas that of IL-10 was reduced in α-syn–stimulated cells of PD compared to HC, suggesting that PD-associated neuroinflammation is not the consequence of the activation of the NLRP3 inflammasome but rather of an imbalance between proinflammatory and anti-inflammatory cytokines.

## Introduction

Parkinson disease (PD) is an age-related neurodegenerative disorder (Rodriguez et al., 2015) characterized by motor (bradykinesia, muscular rigidity, rest tremor, and postural and gait impairment) and non-motor symptoms (including cognitive impairment, psychiatric symptoms, sleep disorders, autonomic dysfunction, pain, and fatigue). All these are thought to be the result, among others, of the death of dopaminergic neurons in the substantia nigra pars compacta (SNpc) of the brain (Ruiperez et al., 2010). The pathological hallmark of PD in the brain is the presence of intracellular Lewy bodies (Spillantini et al., 1997; Phani et al., 2012), the major constituent of which is the misfolded synaptic protein α-synuclein (α-syn). This led to the hypothesis that misfolded α-syn, a cytosolic protein with unclear physiological function (He et al., 2021), could be a key player in disease pathogenesis. α-syn can be found in extracellular biological fluids, including human cerebrospinal fluid (CSF) and blood (Borghi et al., 2000; El-Agnaf et al., 2003), and is released by dying cells (characterized by increased membrane permeability) or is generated by exocytosis from normal cells (Lee, 2008).

The conversion of α-syn from soluble monomers into aggregated, insoluble forms is a key event in the pathogenesis of α-synucleinopathies (Martin et al., 2014). Although it is well-known that α-syn aggregates exert a pathogenic role, causing neuronal death (Yasuda et al., 2013) and neuroinflammation (Zhang et al., 2005), monomeric α-syn was also shown to be endowed with proinflammatory functions (Dohgu et al., 2019). Notably, recent results demonstrated that increased concentrations of α-syn can also be detected in the intestine of PD patients, in whom dysbiosis is often present (Gold et al., 2013; Minato et al., 2017). This observation, together with the experimental evidences that (1) abnormal forms of α-syn can be observed in enteric nerves before they appear in the brain (Corbillé et al., 2016), and (2) abnormal α-syn injected into the wall of the intestine spreads to the vagus nerve (Holmqvist et al., 2014), led to the hypothesis that PD may initiate in the gut.

Neuroinflammation is an important component of PD pathology, as initially suggested by a postmortem study, which demonstrated the presence of activated microglia in the substantia SNpc of PD patients (McGeer et al., 1988). Several clinical and animal studies have confirmed this initial observation, showing that activated microglia and increased levels of inflammatory mediators and reactive oxygen species play an important role in the pathogenesis of PD (Reale et al., 2009a; Gelders et al., 2018).

*In vitro* and *in vivo* experiments demonstrated that, upon exposure to α-syn, microglial cells become activated and release neurotoxic factors, including proinflammatory cytokines (Alvarez-Erviti et al., 2011; Couch et al., 2011), which are also increased in the brains of PD patients (Mogi et al., 1994). Peripheral immune cells also play a key role in PD-associated central nervous system (CNS) damage: peripheral monocyte infiltration in the substantia nigra is, in fact, crucial for neuronal loss induced by the accumulation of α-syn. Additionally, inflammatory cytokines are increased in the serum of PD patients (Brodacki et al., 2008; Reale et al., 2009b), and associations between systemic markers of inflammation and idiopathic PD risk have been reported (Song et al., 2009).

The current body of evidence points to aggregated and post-translationally modified forms of α-syn as a primary cause of microglia activation (Roodveldt et al., 2008; Allen Reish and Standaert, 2015). Taken together, these observations support the hypothesis that α-syn is released early in the disease and, possibly *via* the activation of the inflammasome NLRP3 pathway, stimulates microglia to release proinflammatory cytokines, which are detrimental to dopaminergic neurons. Additional results demonstrated the activation of inflammasomes in the substantia nigra of the brain of PD mouse models and in the CSF of PD patients (Zhang et al., 2016) and showed that stimulation of peripheral monocytes of healthy subjects with aggregated α-syn induces NLRP3-mediated interleukin 1β (IL-1β) production (Codolo et al., 2013). These findings notwithstanding, the role of α-syn in inducing inflammasome activation in PD has not yet been definitively clarified (Heneka et al., 2014), and there is no direct evidence that NLRP3 inflammasome is responsible for microglial activation and neuroinflammation in PD.

Notably, although NLRP3 activation has a beneficial role in promoting inflammation and eliminating infectious pathogens, constitutive inflammasome activation and overproduction of IL-1β and IL-18 were shown to be associated with inflammatory and autoimmune disorders, as well as with neurodegenerative diseases, including Alzheimer disease (AD), multiple sclerosis (MS), and autism (Rubartelli, 2012; Master, 2013; Pollard and Kono, 2013; Saresella et al., 2016a, b; Piancone et al., 2018). To investigate the role of monomeric and aggregated forms of α-syn in PD-associated inflammation, we analyzed NLRP3 inflammasome aggregation and the production of proinflammatory and anti-inflammatory cytokines in PD patients and sex- and age-matched healthy controls (HC) upon stimulation with the different forms of α-syn.

## Materials and Methods

### Patients and Controls

This study was approved by and carried out in accordance with the guidelines of the ethics committee of the Don Gnocchi Foundation and conformed to the Declaration of Helsinki. All participants gave informed consent according to a protocol approved by the local ethics of the Don Gnocchi Foundation.

Fifteen patients (mean age = 76 ± 7 years; range, 63–88 years; 8 females and 7 males; disease duration = 10 ± 3 years) affected by sporadic PD as diagnosed by clinical and laboratory parameters, followed by the Department of Neurology of the Don Gnocchi Foundation in Milan, Italy, and who were undergoing a PD-specific rehabilitation protocol were included in the study. Given that the diagnosis of PD can only be obtained through neuropathology, it has to be considered that the majority but not all the clinical diagnoses of PD are confirmed by neuropathological methods. None of the patients had received immunosuppressive drugs in the year prior to the study period. All the patients were under dopaminergic therapy. Fifteen sex- and age-matched HC (mean age, 70 ± 6 years; range, 62–80 years; 10 females and 5 males) were enrolled as well in the study. The individuals in the control group did not exhibit any signs or symptoms indicative of neurological disease.

### Whole Blood and Plasma Sample Collection and Cell Separation

Thirty milliliters of whole blood was collected in EDTA-containing Vacutainer tubes (Becton Dickinson & Co., Rutherford, NJ, United States). Peripheral blood mononuclear cells (PBMCs) were separated on lymphocyte separation medium (Organon Teknika Corp., Durham, NC, United States) and washed twice in phosphate-buffered saline (PBS). Leukocyte viability was determined using a Bio-Rad TC20 Automated Cell Counter (Bio-Rad, CA, United States). Plasma samples were isolated by cooled centrifugation.

### Cell Cultures: PBMCs

PBMCs (2 × 10^6^) were cultured in RPMI 1640 supplemented with 10% human serum, 2 mM L-glutamine, and 1% penicillin at 37°C in a humidified 5% CO_2_ atmosphere and were (1) unstimulated (medium) or (2) stimulated for 24 h with lipopolysaccharide (LPS) (1 μg/mL) (Sigma-Aldrich, St. Louis, MO) or (3) stimulated for 24 h with monomeric α-syn (5 μM) (Abcam, Cambridge, United Kingdom) or (4) stimulated for 24 h with aggregated α-syn (10 nM) (Abcam, Cambridge, United Kingdom) or (5) primed 2 h with LPS (1 μg/mL) and stimulated for 24 h with monomeric α-syn (5 μM) or (6) primed 2 h with LPS (1 μg/mL) and stimulated for 24 h with aggregated α-syn (10 nM). The aggregated α-syn used in the experiments is a wild-type recombinant human α-syn protein (Abcam, ab2118819) (UniProt: P37840). It is an active protein and is in preformed fibrils, which are generally used to induce α-syn protein aggregation/fibrillization.

### MTT Cell Viability Assay

PBMCs stimulated with 5 μM of monomeric α-syn and/or 10 nM of aggregated α-syn, according to manufacturer’s instructions, were evaluated for vitality with (3-4,5-dimethylthiazol-2-yl-2,5-diphenyl-tretrazolium bromide) the MTT cell viability assay. Briefly, MTT dissolved in PBS was added to the cells (20 μL/well). Cells were incubated at 37°C for 22 h and centrifuged; pellets were dissolved using 100 μL/well of dimethyl sulfoxide (DMSO), and plates were read in a microplate reader using a test wavelength of 550 nm and a reference wavelength of 650 nm. Results were calculated as follows: % cytotoxicity = 100 - (OD test - OD control)/OD control × 100. Cell mortality was comparable <5% in either monomeric and aggregated α-syn stimulation compared to unstimulated condition.

### Enzyme-Linked Immunosorbent Assay

IL-1β, IL-18, caspase-1 p20 subunit (active form), tumor necrosis factor α (TNF-α), IL-6, and IL-10 concentration was determined by sandwich immunoassays according to the manufacturer’s recommendations (Quantikine Elisa Immunoassay; R&D Systems, Minneapolis, MN, United States) in supernatants from unstimulated or stimulated PBMCs. The wells were read on a plate reader (Sunrise, Tecan, Mannedorf, Switzerland), and optical density (OD) of wells was determined at 450/620 nm.

Sensitivity (S) and assay range (AR) were as follows: S: IL-1β = 1 pg/mL, IL-18 = 12.5 pg/mL, caspase-1 = 1.24 pg/mL, IL-10 = 3.9 pg/mL, TNF-α = 6.23 pg/mL, IL-6 = 0.7 pg/mL; AR: IL-1β = 3.91–250 pg/mL, IL-18 = 25.6–1,000 pg/mL, caspase-1 = 6.3–400 pg/mL, IL-10 = 7.8–500 pg/mL, TNF-α = 15.6–1,000 pg/mL, IL-6 = 6.36–600 pg/mL.

The measured absorbance is proportional to the concentration of proteins (IL-1β, IL-18, caspase-1, TNF-α, IL-6, and IL-10) present in the supernatants expressed in pg/mL and calculated by dividing OD measurement generated from the assay by OD cutoff calibrator. All the experiments were performed in triplicate.

### AMNIS FlowSight Imaging Analysis

PBMCs (1 × 10^6^), stimulated as described above, were fixed with 100 μL of Paraformaldehyde (PFA) (1%) (BDH, United Kingdom), permeabilized with 100 μL of saponin (0.1%) (Life Science VWR, Lutterworth, Leicestershire, LE), and stained with PE-antihuman ASC (clone HASC-71, isotype mouse IgG1, Biolegend, San Diego, CA, United States) for 1 h at room temperature; cells were then washed with PBS, centrifuged at 1,500 rpm for 10 min, resuspended in 50 μL of PBS, and examined using the AMNIS FlowSight Imaging Flow Cytometer (Luminex Corporation Austin, TX). Results were analyzed using an analysis software (IDEAS). The IDEAS image analysis software allows quantification of cellular morphology and fluorescence at different cellular localizations by defining specific cellular regions (masks) and mathematical expressions that uses image pixel data or masks (features).

### LPS and I-FABP Concentration in Plasma

Plasma LPS concentration was measured by Hycult Biotech Limulus Amebocyte Lysate (LAL, Hycult Biotechnology, Newark, DE, United States). The assay is a sensitive and specific product available to detect and measure bacterial endotoxin, a fever-producing by-product of gram-negative bacteria commonly known as pyrogen.

Fatty acid–binding protein 2 (FABP2 or I-FABP) concentration was determined by sandwich immunoassay according to the manufacturer’s recommendations (DuoSet Immunoassay; R&D Systems, Minneapolis, MN, United States) in plasma from all the subjects enrolled in the study. Sensitivity (S) and AR were as follows: S: LPS = 0.04 EU/mL, I-FABP = 6.21 pg/mL; AR: LPS = 0.04–10 EU/mL, I-FABP = 15.6–1,000 pg/mL.

### Statistical Analysis

Quantitative data were not normally distributed (Shapiro–Wilk test) and are thus summarized as median and interquartile range. Comparisons within groups were analyzed using a Kruskal–Wallis analysis of variance for each variable. Comparisons between groups were analyzed using a 2-tailed Mann–Whitney *U* test for independent samples. Data analysis was performed using the MedCalc statistical package (MedCalc Software BVBA, Mariakerke, Belgium). *P-*values of less than 0.05 were considered statistically significant. The power of the study, calculated on IL-1β variable, was 76%.

## Results

### ASC-Speck Formation in Monomeric or Aggregated α-Synuclein–Stimulated PBMCs

**FIGURE 1:**
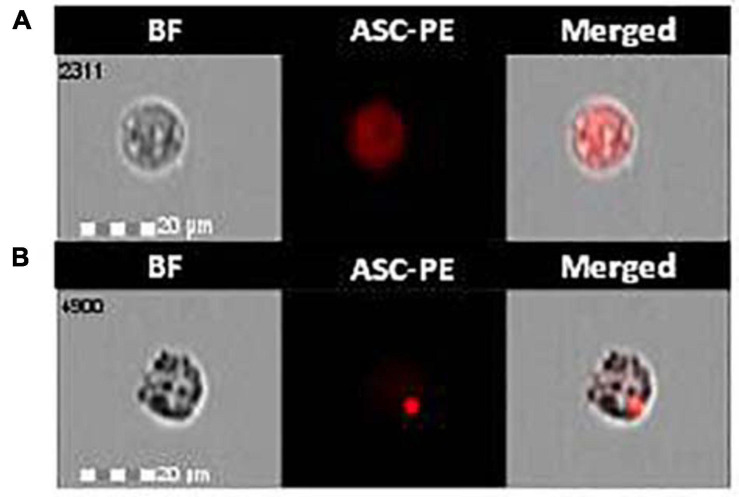
ASC-speck formation in LPS-primed and monomeric or aggregated α-synuclein–stimulated cells of PD and HC subjects. Representative images of ASC-speck formation in LPS-primed and monomeric or aggregated α-synuclein–stimulated PBMCs of PD and HC subjects (**A**, ASC-diffuse; **B**, ASC-speck). The first column shows cells in brightfield (BF), second column shows ASC-PE fluorescence, and third column shows florescence of ASC merged with brightfield (IDEA software). ASC-speck formation was analyzed using an internalization mask that recognizes two different patterns of ASC fluorescence inside cells: diffuse or spot (speck). Threshold mask was used to separate all ASC positive cells population in ASC-speck spot cells or ASC-diffuse cells by the different diameter of the spot area. In ASC-speck cell, the spot showed a small area and high max pixel; conversely in ASC-diffuse cell, the fluorescence showed a large area and low max pixel.

The effect of monomeric and aggregated α-syn on ASC-speck formation was investigated by FlowSight AMNIS analyses in LPS-primed and monomeric or aggregated α-syn–stimulated PBMCs of all patients and controls. Representative images are provided in Figures 1A,B.

**FIGURE 2:**
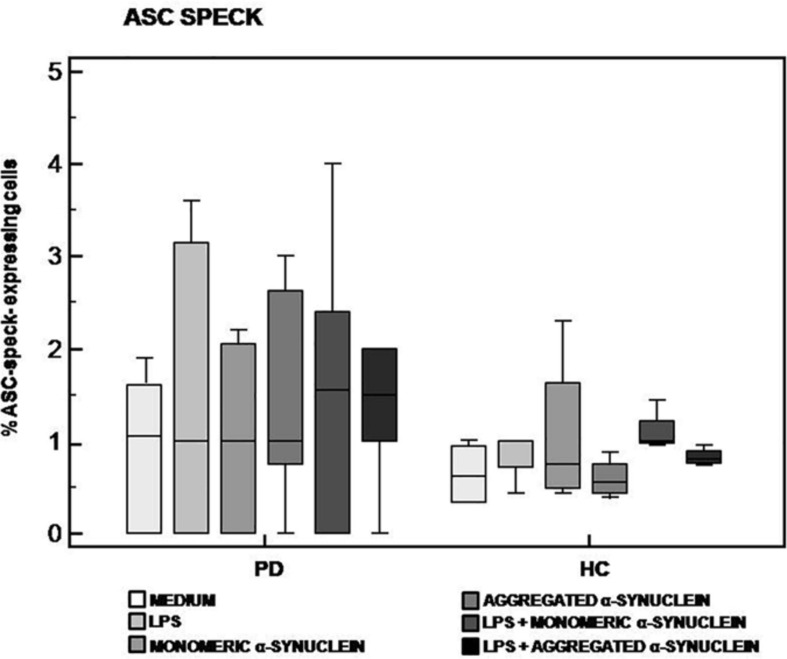
ASC-speck formation in LPS-primed and monomeric or aggregated α-synuclein–stimulated cells of PD and HC subjects. ASC-speck formation in unstimulated, LPS-stimulated, monomeric α-synuclein–stimulated, aggregated α-synuclein–stimulated, LPS-primed and monomeric α-synuclein–stimulated, or LPS-primed and aggregated α-synuclein–stimulated cells of 15 Parkinson disease (PD) patients and 15 healthy controls (HC). Summary results of ASC-speck–positive cells are shown in the bar graphs. The boxes stretch from the 25th to the 75th percentile; the line across the boxes indicates the median values; the lines stretching from the boxes indicate extreme values.

The analysis of ASC-speck formation, i.e., quantification of cells in which fully functional NLRP3 inflammasome complexes are generated, showed that neither monomeric nor aggregated α-syn led to the generation of ASC-specks in cells of either PD or HC (Figure 2). This result indicates that, in these experimental conditions, NLRP3 inflammasome is not activated in PD.

### Caspase-1, IL-1β, and IL-18 Production by LPS-Primed and Monomeric or Aggregated α-Synuclein–Stimulated Cells

Caspase-1, IL-1β, and IL-18, proteins produced upon activation of the NLRP3 inflammasome, were evaluated next in supernatants of unstimulated and of LPS-primed and monomeric or aggregated α-syn–stimulated cells of all individuals enrolled in the study.

**FIGURE 3:**
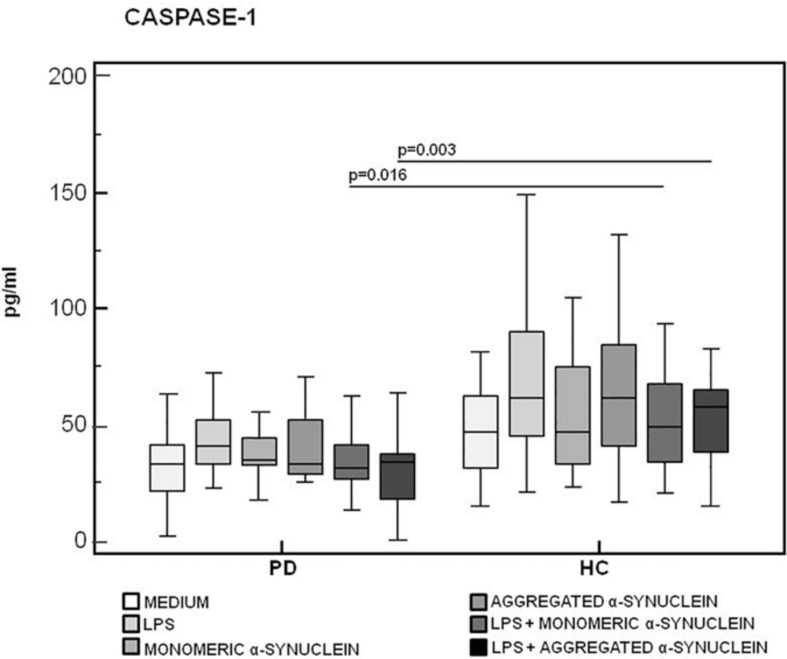
Modulation of active caspase-1 (p20) protein by LPS-primed and monomeric or aggregated α-synuclein–stimulated cells of PD and HC subjects. Caspase-1 production was assessed by enzyme-linked immunosorbent assay in supernatants of unstimulated, LPS-stimulated, monomeric α-synuclein–stimulated, aggregated α-synuclein–stimulated, LPS-primed and monomeric α-synuclein–stimulated, or LPS-primed and aggregated α-synuclein–stimulated cells of 15 Parkinson disease (PD) patients and 15 healthy controls (HC). Summary results are shown in the bar graphs. The boxes stretch from the 25th to the 75th percentile; the line across the boxes indicates the median values; the lines stretching from the boxes indicate extreme values.

**FIGURE 4:**
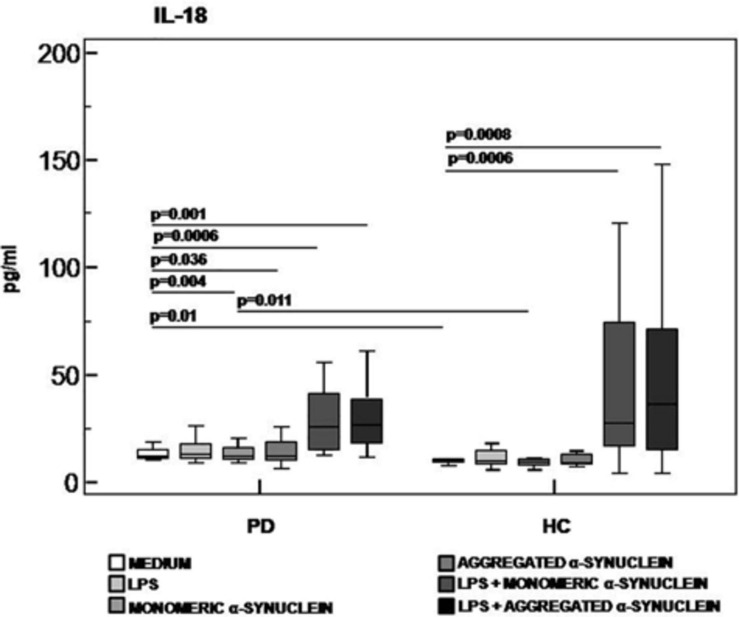
IL-18 inflammasome effector cytokine production in LPS-primed and monomeric or aggregated α-synuclein–stimulated PBMCs of PD and HC subjects. PBMCs of 15 Parkinson disease (PD) patients and 15 healthy controls (HC) were stimulated as follows: unstimulated, LPS-stimulated, monomeric α-synuclein–stimulated, aggregated α-synuclein–stimulated, LPS-primed and monomeric α-synuclein–stimulated, or LPS-primed and aggregated α-synuclein stimulate. IL-1β was quantified in supernatants by enzyme-linked immunosorbent assay. Summary results are shown in the bar graphs. The boxes stretch from the 25th to the 75th percentile; the line across the boxes indicates the median values; the lines stretching from the boxes indicate extreme values.

**FIGURE 5:**
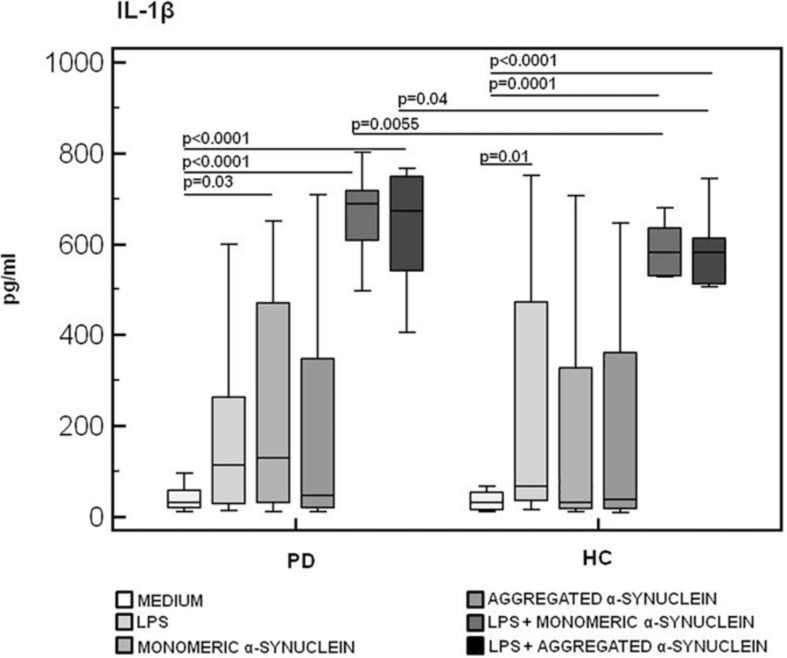
IL-1β inflammasome effector cytokine production in LPS-primed and monomeric or aggregated α-synuclein–stimulated PBMCs of PD and HC subjects. PBMCs of 15 Parkinson disease (PD) patients and 15 healthy controls (HC) were stimulated as follows: unstimulated, LPS-stimulated, monomeric α-synuclein–stimulated, aggregated α-synuclein–stimulated, LPS-primed and monomeric α-synuclein–stimulated, or LPS-primed and aggregated α-synuclein–stimulated. IL-1β was quantified in supernatants by enzyme-linked immunosorbent assay. Summary results are shown in the bar graphs. The boxes stretch from the 25th to the 75th percentile; the line across the boxes indicates the median values; the lines stretching from the boxes indicate extreme values.

Results confirmed that *in vitro* PBMC stimulation with monomeric or aggregated α-syn does not result in the activation of the NLRP3 inflammasome. Thus, (1) caspase-1 (p20 subunit) production was similar in all conditions in PD and HC (Figure 3), and (2) IL-18 production was comparable in HC and PD cells that were stimulated either with monomeric (median *PD* = 26.9 pg/mL, *HC* = 28.06 pg/mL) or aggregated (median *PD* = 30.9 pg/mL, *HC* = 36.2 pg/mL) α-syn (Figure 4). In contrast with these results, IL-1β production was significantly increased in LPS-primed cells of PD that were stimulated with either monomeric (median *PD* = 675.8 pg/mL, *HC* = 570.4 pg/mL) (*p* = 0.005) or aggregated (median *PD* = 666.15 pg/mL, *HC* = 571 pg/mL) (*p* = 0.04) α-syn (Figure 5).

### IL-6, TNF-α, and IL-10 Production in LPS-Primed and Monomeric or Aggregated α-Synuclein–Stimulated PBMCs of PD and HC Subjects

**FIGURE 6:**
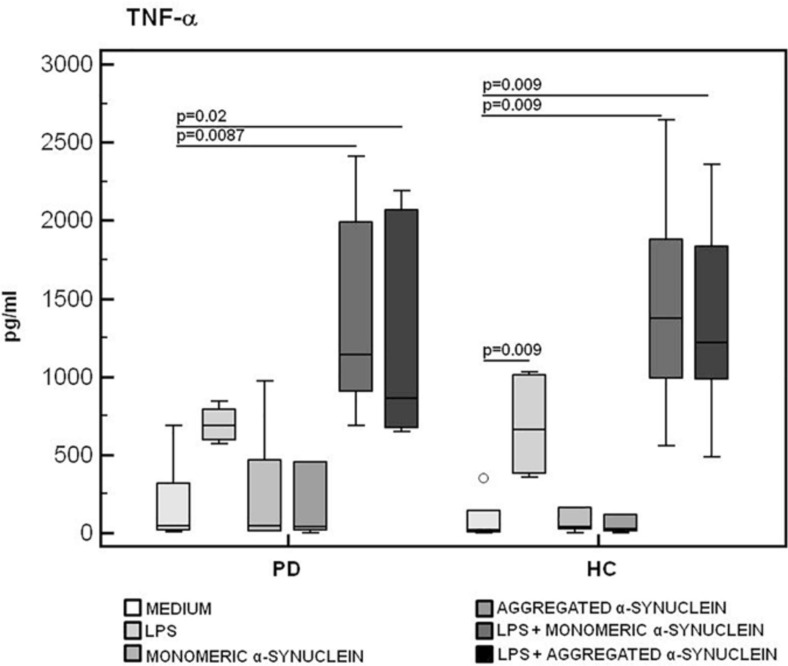
TNF-α proinflammatory cytokine production by monomeric or aggregated α-synuclein–stimulated cells of PD and HC subjects. TNF-α concentration was evaluated by enzyme-linked immunosorbent assay in supernatants of unstimulated, LPS-stimulated, monomeric α-synuclein–stimulated, aggregated α-synuclein–stimulated, LPS-primed and monomeric α-synuclein–stimulated, or LPS-primed and aggregated α-synuclein–stimulated cells of 15 Parkinson disease (PD) patients and 15 healthy controls (HC). Summary results are shown in the bar graphs. The boxes stretch from the 25th to the 75th percentile; the line across the boxes indicates the median values; the lines stretching from the boxes indicate extreme values.

**FIGURE 7:**
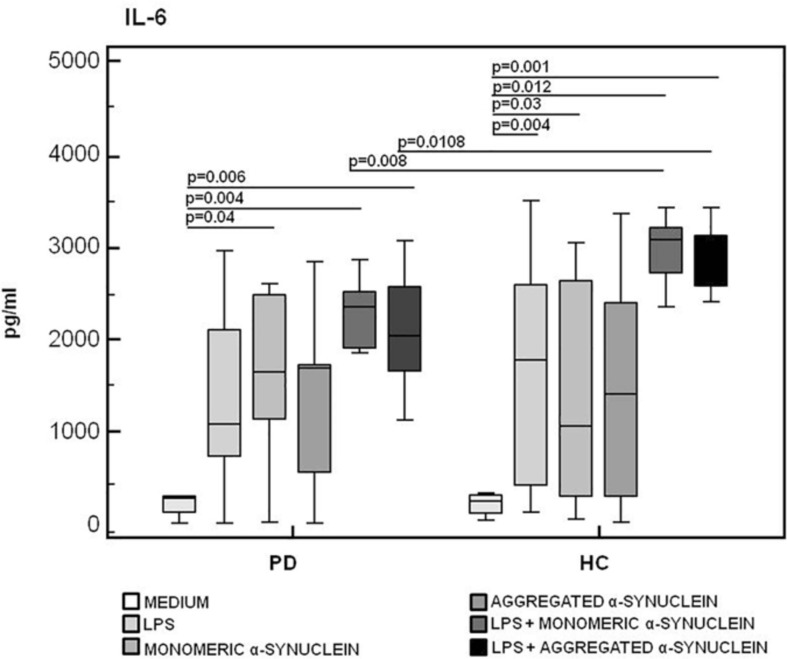
IL-6 proinflammatory cytokine production by monomeric or aggregated α- synuclein–stimulated cells of PD and HC subjects. IL-β was quantified by enzyme-linked immunosorbent assay in supernatants of Parkinson disease (PD, *n* = 15) and healthy controls (HC, *n* = 15). PBMCs stimulated as follows: unstimulated, LPS-stimulated, monomeric α-synuclein–stimulated, aggregated α-synuclein–stimulated, LPS-primed and monomeric α-synuclein–stimulated, or LPS-primed and aggregated α-synuclein–stimulated. Summary results are shown in the bar graphs. The boxes stretch from the 25th to the 75th percentile; the line across the boxes indicates the median values; the lines stretching from the boxes indicate extreme values.

The production of the proinflammatory cytokines TNF-α and IL-6 and of the anti-inflammatory cytokine IL-10 was finally analyzed in LPS-primed and monomeric or aggregated α-syn–stimulated PBMCs. Results showed that, whereas no differences were observed in TNF-α (Figure 6), IL-6 production was significantly increased in HC compared to PD in PBMCs stimulated with either monomeric (*HC* = 3,071.2 pg/mL; *PD* = 2,341.5 pg/mL) (*p* = 0.008) or aggregated (*HC* = 2,821.3 pg/mL; *PD* = 2,031.2 pg/mL) (*p* = 0.01) α-syn (Figure 7).

**FIGURE 8:**
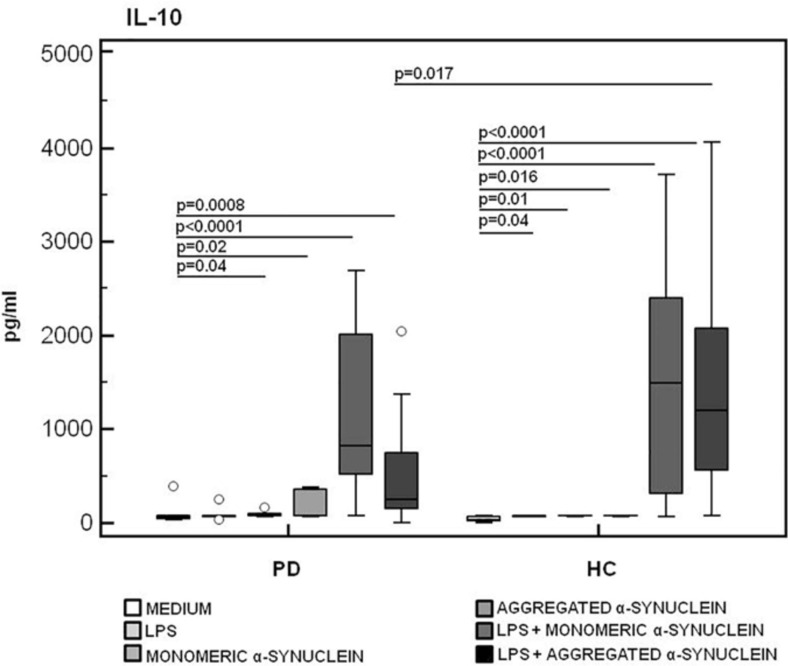
IL-10 anti-inflammatory cytokine production by monomeric or aggregated α-synuclein–stimulated cells of PD and HC subjects. IL-10 production was analyzed in supernatants from PBMCs cultures of Parkinson disease (PD, *n* = 15) and healthy controls (HC, *n* = 15). PBMCs were unstimulated, LPS-stimulated, monomeric α-synuclein–stimulated, aggregated α-synuclein–stimulated, LPS-primed and monomeric α-synuclein–stimulated, or LPS-primed and aggregated α-synuclein–stimulated. Summary results are shown in the bar graphs. The boxes stretch from the 25th to the 75th percentile; the line across the boxes indicates the median values; the lines stretching from the boxes indicate extreme values.

In contrast with these results, the production of IL-10, a potent anti-inflammatory protein, was significantly reduced in LPS-primed and aggregated α-syn–stimulated PBMCs of PD compared to the values observed in HC (*HC* = 1,195.8 pg/mL, *PD* = 268.7 pg/mL) (*p* = 0.017) (Figure 8).

### I-FABP and LPS Plasma Quantification

**FIGURE 9:**
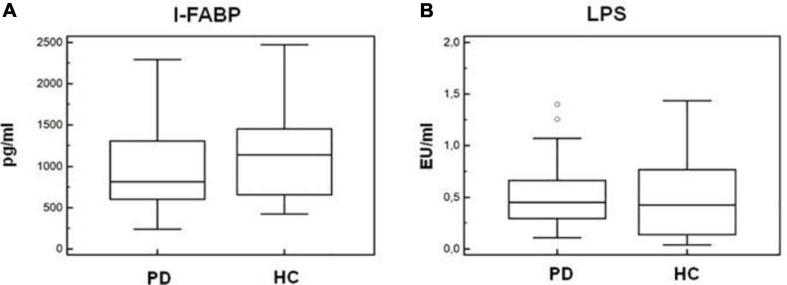
Plasma I-FABP and LPS concentration. Microbial translocation and gut barrier permeability markers. I-FABP **(A)** and LPS **(B)** concentration (pg/mL and EU/mL, respectively) in plasma of Parkinson disease patients (PD, *n* = 15) and healthy controls (HC, *n* = 15). Summary results are shown in the bar graphs. The boxes stretch from the 25th to the 75th percentile; the line across the boxes indicates the median values; the lines stretching from the boxes indicate extreme values. Outside values are displayed as separate points.

As α-syn is also present in the gastrointestinal (GI) tract in the enteric nervous system, and gut microbiota alterations were suggested to be present in PD, we verified whether alterations of permeability could help justify the inflammatory milieu seen in PD. To this end, we quantified I-FABP and LPS plasma concentration in all the individuals enrolled in the study. Results indicated that plasma concentration of these two proteins was similar in HC and PD patients (I-FABP: *PD* = 814 pg/mL, *HC* = 1,140 pg/mL; LPS: *PD* = 0.45 EU/mL, *HC* = 0.42 EU/mL) (Figures 9A,B).

## Discussion

Neurodegenerative diseases are chronic progressive disorders that occur in the CNS, are characterized by the loss of neuronal structure and function, and are associated with inflammation and immune abnormalities. Neuroinflammation accompanies the progression of PD and involves α-syn (Hirsch et al., 2013; Dohgu et al., 2019) as it can be secreted from neurons and transferred to nearby cells including astrocytes and oligodendrocytes (Desplats et al., 2009) and microglial activation, which can be observed in postmortem brains of patients with a recent diagnosis of PD (Ouchi et al., 2005), as well as in brains of patients who died decades after disease onset (McGeer et al., 1988). In addition to microglia activation, neuroinflammation is characterized by monocyte recruitment from peripheral blood into the CSF and the production of proinflammatory cytokines including IL-1β.

Over the past 5 years, the involvement of the inflammasome complex has been shown in the pathogenesis of neurodegenerative disease including AD and MS, as well as in neurodevelopmental pathologies, such as autism spectrum disorders (ASDs) (Saresella et al., 2016a, b; Piancone et al., 2018). In PD, activation of the inflammasome was shown in mouse models and in the CSF of PD patients (Zhang et al., 2016), and stimulation of peripheral monocytes of healthy subjects with aggregated α-syn was demonstrated to induce NLRP3-mediated IL-1β production (Codolo et al., 2013). These findings notwithstanding, it is important to underline that no direct evidence that NLRP3 inflammasome is responsible for microglial activation and neuroinflammation is available in PD.

Given these premises, we decided to investigate whether monomeric or aggregated α-syn can drive NLRP3 inflammasome activation in PD. Results showed this not to be the case as neither the assembly of NLRP3 inflammasome proteins to create a functionally active complex, nor the production of NLRP3-derived cytokines could be detected in our experimental model. Indeed, ASC–caspase-1 specks, whose production is fundamental for NLRP3 inflammasome activation (Stutz et al., 2013; He et al., 2016), were comparable between PD and HC subjects.

Notably, though, the generation of IL-1β, a proinflammatory cytokine that can be produced upon the activation of the NLRP3 inflammasome but can be also independently generated, was greatly augmented by α-syn–stimulated cells of PD individuals and was accompanied by a significantly increased production of IL-6. A similar response could be observed in α-syn–stimulated cells from HC, in whom an increase of caspase-1 activation, even if not statistically significant compared to the unstimulated condition, can be observed; nevertheless, only in PD patients this response is associated with a drastic reduction of IL-10 generation. Taken together, these results indicate a proinflammatory milieu is triggered in PD by α-syn independently of the activation of the NLRP3 inflammasome.

These results are in line with previous observations indicating that increased levels of proinflammatory cytokines are observed in CSF and serum of PD patients and that production of such cytokines is augmented by activated microglia of PD individuals (Brodacki et al., 2008; Reale et al., 2009a, b; Chao et al., 2014). Notably, data herein showing that IL-10 generation is potent in α-syn–stimulated cells of HC, but is greatly reduced in those of PD, allow speculating that an IL-10–mediated anti-inflammatory loop is active in physiological conditions and is lost upon development of PD. Our data, on the other hand, are in disagreement with older results suggesting that α-syn does induce the activation of the NLRP3 inflammasome (Codolo et al., 2013). This discrepancy could be explained by the observation that we could perform a direct measurement of NLRP3 activation by quantifying the aggregation of inflammasome constitutive proteins, as well as caspase-1 and IL-18 production, whereas Codolo et al. (2013) inferred NLRP3 activation by measuring IL-1β production, the standard method used when that article was published.

Recent results suggested that gut microbiota dysbiosis is present in PD patients (Minato et al., 2017), in whom the intestinal concentration of α-syn is increased as well (Gold et al., 2013), possibly resulting in GI dysfunction (Sánchez-Ferro et al., 2015). Lewy body pathology is also observed in the enteric nervous system in the GI tract, suggesting that α-syn pathology in PD may start in the GI tract. On this basis, and because alterations of the GI permeability were shown to play an important proinflammatory role in chronic diseases (Brenchley et al., 2006; Steele et al., 2014), we analyzed GI permeability by measuring the concentration of plasma I-FABP and LPS. I-FABP is a small cytosolic protein present in minimal amounts in the plasma of healthy individuals, but it rapidly increases when cell membrane integrity is damaged (Lieberman et al., 1997; Brenchley et al., 2006). LPS translocates from the intestinal lumen to peripheral circulation when the integrity of the GI barrier is altered. Results showed that plasma levels of LPS and I-FABP were comparable in PD and HC, suggesting that the permeability of the GI barrier is not compromised in PD and indicating that inflammation in PD is not likely to be driven by GI tract alterations.

To summarize, our data suggest that PD-associated neuroinflammation is not driven by the activation of the NLRP3 inflammasome and is thus different from the neuroinflammatory scenario that characterizes AD, MS, and ASD and is indeed supported by the NLRP3 inflammasome. If this is the case, how is PD-associated neuroinflammation generated? It is interesting to observe that α-syn was shown to induce the upregulation of TLRs on microglia (Béraud et al., 2011); such upregulation, together with the skewing in cytokine production by α-syn–stimulated cells that we describe herein, could play a role in this phenomenon. Further analyses in ampler cohorts of patients will be necessary to further clarify this aspect of PD. A final note of cautions stems from the observation that these are results originated by analyzing peripheral blood cells; the origin of neuroinflammation in the brain could indeed be different.

## Data Availability Statement

The raw data supporting the conclusions of this article will be made available by the authors, without undue reservation, to any qualified researcher.

## Ethics Statement

The studies involving human participants were reviewed and approved by the Ethic Committee of the Don Gnocchi Foundation. The patients/participants provided their written informed consent to participate in this study.

## Author Contributions

FP, MS, and MC conceived and designed the research. FP, IM, FL, and MS performed the experiments. MM and JN were responsible for the clinical cohorts of patients. FP and MC analyzed the data and prepared the manuscript. All authors reviewed and approved the final manuscript.

## Conflict of Interest

The authors declare that the research was conducted in the absence of any commercial or financial relationships that could be construed as a potential conflict of interest.
